# Comparing prediction accuracy for 30-day readmission following primary total knee arthroplasty: the ACS-NSQIP risk calculator versus a novel artificial neural network model

**DOI:** 10.1186/s43019-024-00256-z

**Published:** 2025-01-13

**Authors:** Anirudh Buddhiraju, Michelle Riyo Shimizu, Tony Lin-Wei Chen, Henry Hojoon Seo, Blake M. Bacevich, Pengwei Xiao, Young-Min Kwon

**Affiliations:** Bioengineering Laboratory, Department of Orthopedic Surgery, Massachusetts General Hospital, Harvard Medical School, 55 Fruit Street, Boston, MA 02114 USA

**Keywords:** Readmissions, Total knee arthroplasty, Risk assessments, Deep learning, Clinical decision support

## Abstract

**Background:**

Unplanned readmission, a measure of surgical quality, occurs after 4.8% of primary total knee arthroplasties (TKA). Although the prediction of individualized readmission risk may inform appropriate preoperative interventions, current predictive models, such as the American College of Surgeons National Surgical Quality Improvement Program (ACS-NSQIP) surgical risk calculator (SRC), have limited utility. This study aims to compare the predictive accuracy of the SRC with a novel artificial neural network (ANN) algorithm for 30-day readmission after primary TKA, using the same set of clinical variables from a large national database.

**Methods:**

Patients undergoing primary TKA between 2013 and 2020 were identified from the ACS-NSQIP database and randomly stratified into training and validation cohorts. The ANN was developed using data from the training cohort with fivefold cross-validation performed five times. ANN and SRC performance were subsequently evaluated in the distinct validation cohort, and predictive performance was compared on the basis of discrimination, calibration, accuracy, and clinical utility.

**Results:**

The overall cohort consisted of 365,394 patients (training_N_ = 362,559; validation_N_ = 2835), with 11,392 (3.1%) readmitted within 30 days. While the ANN demonstrated good discrimination and calibration (area under the curve (AUC)_ANN_ = 0.72, slope = 1.32, intercept = −0.09) in the validation cohort, the SRC demonstrated poor discrimination (AUC_SRC_ = 0.55) and underestimated readmission risk (slope = −0.21, intercept = 0.04). Although both models possessed similar accuracy (Brier score: ANN = 0.03; SRC = 0.02), only the ANN demonstrated a higher net benefit than intervening in all or no patients on the decision curve analysis. The strongest predictors of readmission were body mass index (> 33.5 kg/m^2^), age (> 69 years), and male sex.

**Conclusions:**

This study demonstrates the superior predictive ability and potential clinical utility of the ANN over the conventional SRC when constrained to the same variables. By identifying the most important predictors of readmission following TKA, our findings may assist in the development of novel clinical decision support tools, potentially improving preoperative counseling and postoperative monitoring practices in at-risk patients.

## Background

Primary total knee arthroplasty (TKA) is a common surgery for end-stage knee osteoarthritis that is expected to increase in prevalence to 1.28 million procedures annually by 2030 [1]. As a result, the overall number of associated complications is also expected to increase. Postoperative complications, including readmission, are not uncommon following TKA, with recent studies reporting a 30-day readmission rate of 4.8% [2, 3]. Readmission following primary TKA is a significant contributor to the overall episode of care cost, with a study reporting a median expenditure of $6753 per episode [4, 5]. Unplanned readmission is also known to affect patient satisfaction and subsequent functional recovery after surgery [6, 7]. Identifying patients at risk of readmission following TKA may help mitigate the incidence of suboptimal outcomes and reduce healthcare costs while improving patient satisfaction [8].

The American College of Surgeons National Surgical Quality Improvement Program (ACS NSQIP) Surgical Risk Calculator (SRC) is a publicly available tool developed using 2016–2020 NSQIP data that offers risk estimates of various postoperative outcomes using a predetermined set of input variables related to patient factors and the operative procedure [9]. Although the SRC has demonstrated reasonable utility in assessing the patient-specific risk of postoperative complications, studies have shown inconsistencies in its accuracy for orthopedic procedures [10–13]. In particular, the SRC has shown poor performance in predicting readmission risk following TKA [14, 15]. Machine learning (ML) models have gained a growing interest over recent years as an accurate risk-prediction tool. Many studies across various fields, including arthroplasty, have endorsed the predictive performance of ML algorithms for postoperative outcomes [16–18]. The novelty of ML models is their ability to effectively analyze large quantities of patient data to identify nonlinear patterns between various risk factors and prediction targets that cannot be identified using conventional statistical methods [19]. ML models trained on institutional or national databases have previously been successfully applied to predict unplanned readmission after primary TKA [20]. The integration of these models in a clinical setting may aid in patient optimization and monitoring, allowing surgeons to individualize patient care based on predicted outcomes.

Despite their promise, however, ML models are limited by the databases selected for their development. Consequently, there has been increasing concern for the generalizability of ML models developed using institutional databases [21]. The development of an ML model using a nationwide dataset such as ACS-NSQIP would allow for consistent interpretation across various hospital systems and patient populations in the USA. Recent evidence comparing the predictive performance of both tools suggests that ML models may possess greater predictive performance than the SRC. Liu et al. found that an XGBoost algorithm trained on the ACS-NSQIP database demonstrated a higher accuracy than the SRC across all surgeries and postoperative outcomes [22]. However, there is a paucity of published data that evaluate the difference in the predictive performance of both methods for 30-day readmission following primary TKA. Therefore, we aim to compare the performance of the ACS NSQIP SRC to an ML model in predicting 30-day readmission after primary total knee arthroplasty, using the same set of variables from a large national database.

## Methods

### Data source

This study was determined to be exempt from review by the institutional review board (IRB), as it only utilizes deidentified patient data made available through the American College of Surgeons National Surgical Quality Improvement Program (ACS-NSQIP) database. All adult patients who underwent primary total knee arthroplasty (TKA) between 2013 and 2020 in the USA were identified using the current procedural terminology (CPT) code 27447 (arthroplasty, knee, condyle, and plateau; medial and lateral compartments with or without patella resurfacing (total knee arthroplasty)) from the ACS-NSQIP database. The ACS-NSQIP records over 150 variables routinely available in electronic health records, including demographic, preoperative, intraoperative, and postoperative data. Patients who underwent bilateral TKA, had missing data, or were under the age of 18 at the time of surgery were excluded from the analysis. The publicly available SRC was developed by the American College of Surgeons using NSQIP data between 2016 and 2020.

### Outcomes and variables

The outcome of interest was the occurrence of 30-day readmission following primary TKA, which was also the basis for cohort stratification. To maintain consistency of comparison, only variables included in the SRC were utilized to develop the ML model. Artificial neural network (ANN) was selected for development in accordance with its excellent predictive performance in the existing literature [16, 18, 23]. The following demographic variables and comorbidities were included in both the SRC and ANN models: age, sex, body mass index (BMI), American Society of Anesthesiologists (ASA) class, functional health status, steroid use, ascites, ventilator dependence, malignancy, diabetes, hypertension, congestive heart failure, dyspnea, smoking status, chronic obstructive pulmonary disease, dialysis dependence, and renal failure.

### Model development, assessment, and comparison

The ACS-NSQIP data were divided into two datasets, the training and validation datasets, by randomized stratification, which were used for model development and validation, respectively. The ANN model was developed using the training dataset, during which the model was optimized by hyperparameter tuning followed by fivefold cross-validation performed five times. The predictive performance of the ANN model was then assessed in the distinct validation dataset. Subsequently, individual patient data from the validation dataset was manually input into the publicly available SRC to evaluate its predictive performance [9]. Both the ANN and the ACS NSQIP SRC models were then evaluated and compared on the basis of discrimination, calibration, accuracy, and decision curve analyses as previously established in the literature [20, 24, 25]. Discrimination describes the model’s ability to distinguish between those who develop the outcome and those who do not and is measured by the area under the receiver operator characteristic curve. An area under the curve (AUC) score above 0.8 demonstrates excellent discrimination [26]. The calibration plot is composed of the slope and intercept and measures predictive model calibration. An ideal calibration plot exhibits a slope of 1 and an intercept of 0 [27]. Alternatively, a slope and intercept that deviate from the above values indicate poor model calibration. Brier score represents the accuracy and validity of both SRC and ML models. A Brier score of 0 represents perfect predictive accuracy, while a value of 1 indicates a perfectly inaccurate model [28]. Decision curve analysis evaluates the model’s theoretical net benefit when used to assist clinical decision-making compared with the conventional strategies of intervening in all or no patients [29].

### Data analyses

All analyses were performed using Anaconda (Anaconda Inc., Austin, Texas, USA), Python (Python Software Foundation, Wilmington, Delaware, USA), and SPSS (SPSS Version 18.0, IBM Corp., Armonk, New York, USA). On the basis of the data distribution assessed using the Shapiro–Wilk test, continuous group descriptives are reported as mean (± SD) or median (± IQR), whereas categorical variables are reported as percentages.

## Results

### Patient cohort

A total of 365,394 patients who underwent a primary TKA in the USA were selected from the NSQIP dataset, of whom 11,392 had a record of unplanned readmission within 30 days after a primary TKA. The training dataset consisted of 362,559 patients, and 2835 patients composed the validation dataset, which was limited by the number of manual entries input into the SRC. Readmitted patients were, on average, significantly older (69 versus 67 years; *p* < 0.001) and with a higher BMI (33.51 versus 33.09; *p* < 0.001) than the non-readmitted patients. Men accounted for a greater proportion of patients in both the readmission (56.2%) and non-readmission groups (61.8%). There were a greater number of patients who were current smokers (10.1%) in the readmission group compared with non-smokers (8.1%). A significant difference in the prevalence of comorbidities between the two cohorts was also observed. The readmission cohort had a significantly higher proportion of patients with diabetes, dependence on ventilator use, steroid use, congestive heart failure, systemic inflammatory response syndrome, and moderate dyspnea. In contrast, a lower percentage of readmitted patients had a history of hypertension prior to undergoing surgery (26.5% versus 35.8%; *p* < 0.001). There was a significant difference in ASA classification between the two cohorts (*p* < 0.001, Table 1).

**Table 1:** The baseline parameters of the overall primary total knee arthroplasty patient cohort

Parameter		Non-readmitted (*n* = 354,002)	Readmitted (*n* = 11,392)	*p*-Value
Sociodemographics				
Age (years)		66.8 ± 9.3	68.9 ± 10.0	**< 0.001**
BMI (kg/m^2^)		33.1 ± 6.7	33.5 ± 7.3	**< 0.001**
Sex (men, %)		61.8	56.2	**< 0.001**
Sex (women, %)		38.2	43.8	**< 0.001**
Comorbidities				
Smoking (%)		8.1	10.1	**< 0.001**
Hypertension (%)		35.8	26.5	**< 0.001**
Diabetes (%)		18.2	23.7	**< 0.001**
Preoperative steroid use (%)		3.6	5.6	**< 0.001**
Congestive heart failure (%)		0.3	1.1	**< 0.001**
Systemic sepsis				
None (%)		99.8	99.7	**0.007**
SIRS (%)		0.2	0.3	
Sepsis or septic shock (%)		0.01	0.0	
Dyspnea				
None (%)		94.5	90.1	**< 0.001**
Moderate exertion (%)		5.4	9.3	
At rest (%)		0.2	0.6	
Functional status				
Independent (%)		99.0	97.4	**< 0.001**
Partially or totally dependent (%)		1.1	2.6	
ASA classification				
1 (%)		47.9	33.4	**< 0.001**
2 (%)		50.2	65.6	
3 (%)		1.8	1.0	
4 or above (%)		0.01	0.0	

BMI: body mass index; SIRS: systemic inflammatory response syndrome; ASA: American Society of Anesthesiologists. Bold values represent statistical significance, with *p* < 0.05

### Model assessment and comparison

**Fig. 1:**
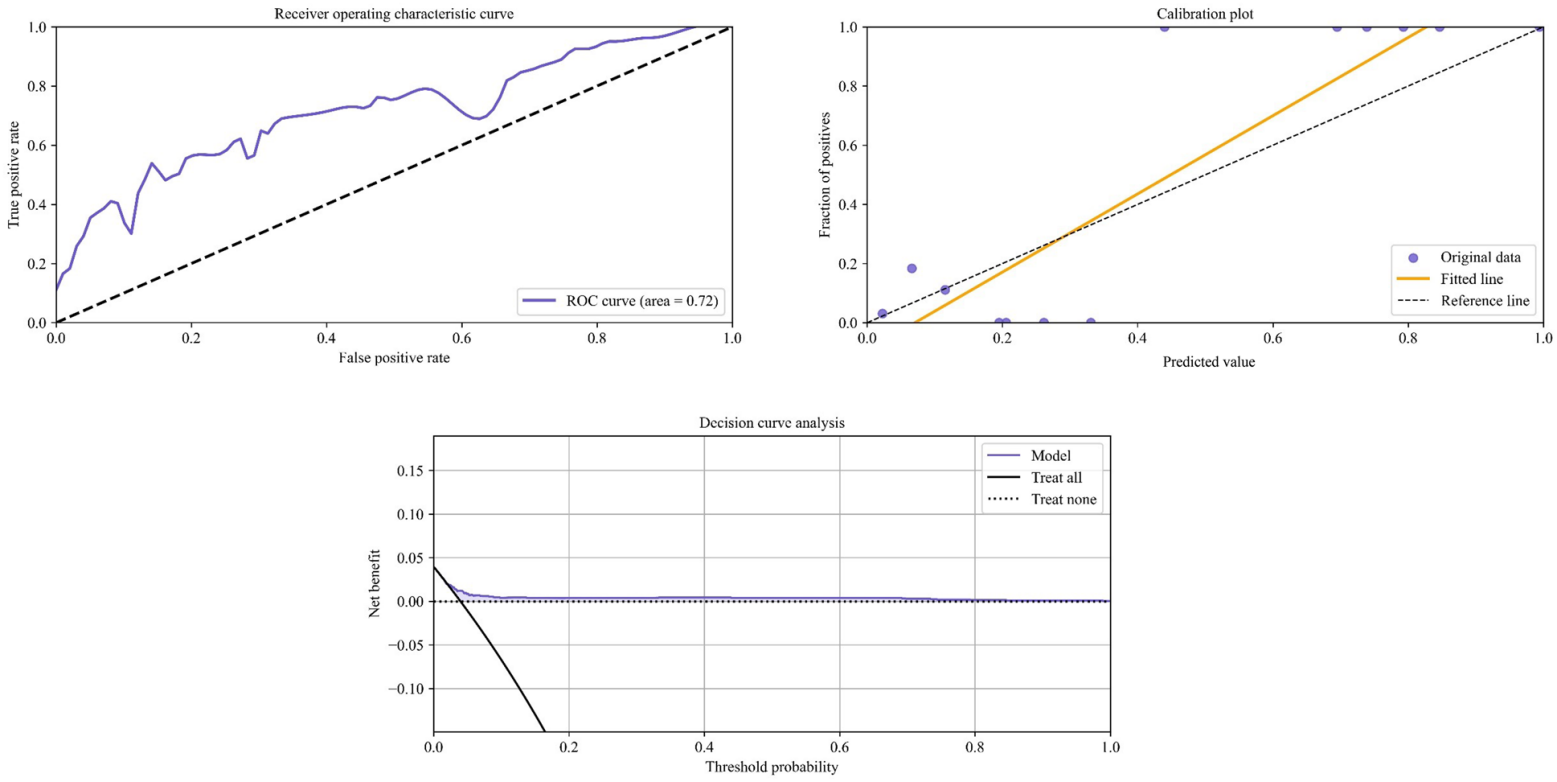
Receiver operating characteristic curve, calibration plot, and decision curve of the artificial neural network model in predicting 30-day readmission rates following primary total knee arthroplasty

**Fig. 2:**
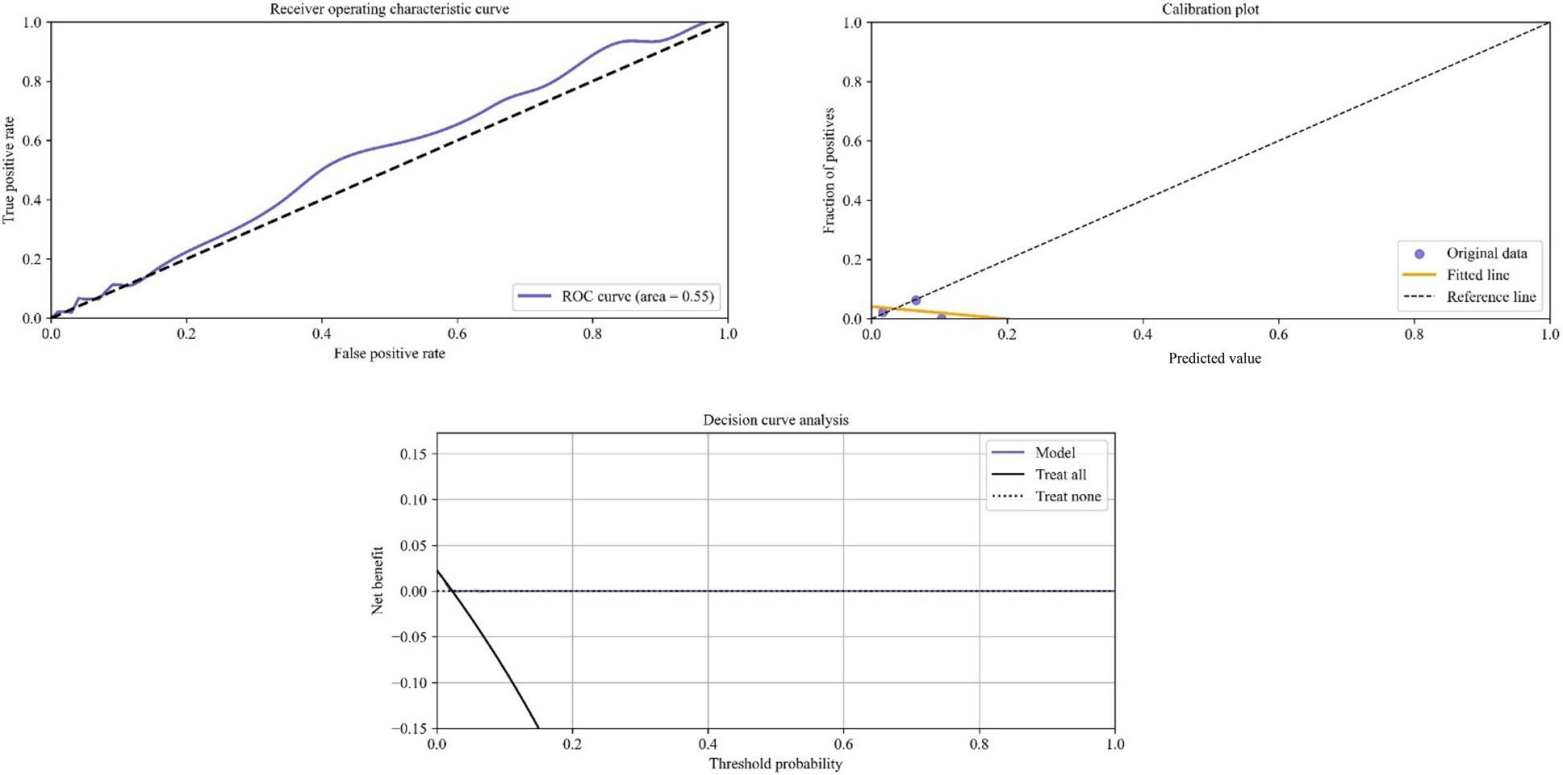
Receiver operating characteristic curve, calibration plot, and decision curve of the ACS-NSQIP surgical risk calculator in predicting 30-day readmission rates following primary total knee arthroplasty

The ANN demonstrated good discrimination in the validation dataset with the AUC_ANN_ = 0.72 (Fig. 1). In contrast, SRC demonstrated poor discrimination with the same dataset with the AUC_SRC_ = 0.55 (Fig. 2). The ANN algorithm demonstrated improved calibration performance comparatively, with a slope of 1.32 and an intercept of −0.09. Conversely, the SRC calibration plot exhibited a slope of −0.21 and an intercept of 0.04. The Brier score of the ANN (0.034) and that of the SRC (0.022) were found to be similar, implying similar predictive accuracy (Table 2). On performing decision curve analysis, however, only the ANN model demonstrated a higher net benefit in assisting clinical decision-making compared with implementing default strategies such as intervening in all or no patients (Fig. 1).

**Table 2:** Discrimination and calibration of the artificial neural network and the ACS-NSQIP surgical risk calculator for the prediction of 30-day readmission following primary total knee arthroplasty

Metric	Artificial neural network	ACS risk calculator
AUC	0.72	0.55
Slope (calibration plot)	1.32	− 0.21
Intercept (calibration plot)	− 0.09	0.04
Brier score	0.034	0.022

AUC: Area under the receiver operating characteristic curve; ACS: American College of Surgeons.

### Model interpretation

**Fig. 3:**
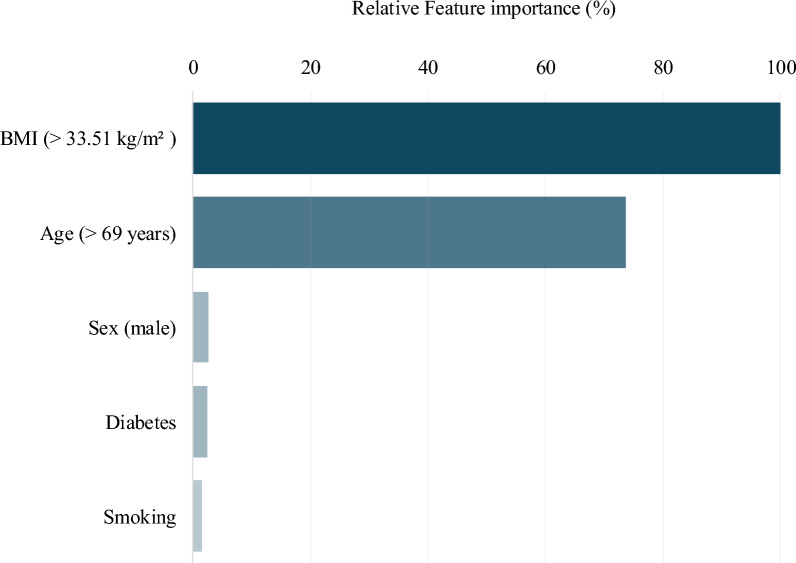
Global predictor importance plot illustrating the relative importance of each predictor in the ANN model for determining the risk of readmission following primary total knee arthroplasty

The three most powerful predictors of 30-day readmission were identified by the ANN model as body mass index (BMI; > 33.51 kg/m^2^), age (> 69 years), and male sex (Fig. 3). Of these, a BMI greater than 33.51 kg/m^2^ was the most important predictor of being readmitted within 30 days postoperatively. Similarly, age over 69 years and male sex were associated with an increased risk of 30-day readmission.

## Discussion

This study found that the ANN model demonstrated higher discriminatory ability and superior calibration compared with the ACS-NSQIP SRC when developed using the same clinical variables. Although both models were found to possess similar predictive accuracy, with similar Brier scores, only the ANN model demonstrated a potential clinical utility on performing decision curve analysis. The ANN model also identified the strongest risk factors for readmission following total knee arthroplasty as having a body mass index greater than 34, age greater than 69 years, and male sex. By attributing contributory weights to previously known risk factors, the findings of this study may assist in prioritizing patient-specific preoperative optimization and counseling, potentially decreasing the occurrence of unplanned 30-day readmission following primary total knee arthroplasty.

The ACS-NSQIP surgical risk calculator is a validated and publicly available tool using 2016–2020 NSQIP data to estimate surgical risk. Previous studies, however, reported mixed performance of the SRC in predicting patient-specific risk following orthopedic surgery, particularly for certain postoperative outcomes such as readmission, where its estimates have been shown to be inadequate [10–15, 30]. An accurate estimation of the risk of developing postoperative complications is essential in reducing the overall incidence of these adverse events after surgery. One possible reason for the relatively lower accuracy of the SRC may be the utilization of logistic regression models for risk prediction. Several studies across various disciplines of healthcare have previously reported on the improved predictive ability of machine learning models over traditional statistical techniques such as logistic regression [31–34]. Another potential factor contributing to the SRC’s performance may be owing to the dataset composition used during validation. While the SRC was developed using 2016–2020 NSQIP data, the exact dataset used remains unknown. As a result, the validation dataset may differ sufficiently from the development dataset, categorizing the SRC as a type 3 model and possibly explaining its poorer overall performance [35]. Conversely, the ANN would be classified as a type 2a model owing to the random stratification of data used for training and validation. However, it remains that the AUC achieved by the SRC was 0.55, implying that it is only marginally better than chance at predicting readmission following TKA. Several prior studies in orthopedic surgery have extolled the predictive capabilities of machine learning algorithms for postoperative outcomes after surgery [16, 18, 20, 23, 25]. Furthermore, with the recent emergence of deep learning, a subfield of machine learning, the capabilities of predictive algorithms such as the ANN are only expected to improve further. A recent review of multiscale deep learning applications revealed the promise of these upcoming algorithms in improving predictive accuracy over existing machine learning approaches [36]. As ANN forms one of the foundational components of deep learning, the ML algorithm developed in this study may benefit from the inclusion of additional input parameters to improve model performance. As such, existing machine learning applications in orthopedics have been shown to demonstrate equivalent [37, 38], if not better, predictive capabilities compared with all other existing alternatives [39], thereby supporting the findings of the current study.

Conversely, the recent interest in the development of novel predictive machine learning has also resulted in the proliferation of several inadequately validated algorithms [40, 41]. Without appropriate validation, the application of the model is limited to the dataset on which it was developed, resulting in poor predictive performance in different data contexts [21, 41]. Recent studies emphasize the importance of incorporating geographically diverse patient data during model development and validation to improve predictive generalizability and applicability across different clinical settings [24, 42]. To achieve this, we trained the model using a national dataset from the ACS-NSQIP, which aggregates patient data from multiple health centers across the USA. Additionally, the predictive performance of the ANN model was validated on a distinct dataset, which was also used to validate SRC predictions, allowing for consistency in comparison. The ANN was found to possess higher discrimination capabilities (AUC_ANN_ = 0.72; AUC_SRC_ = 0.55) and better calibration (slope = 1.32, intercept = −0.09) compared with the SRC (slope = −0.21, intercept = 0.04). The SRC’s poorer performance (AUC = 0.55) may also be in part due to potential differences in validation strategies between the models. While the SRC was developed using 2016–2020 NSQIP data, the exact subset used remains unclear. If the validation dataset differed temporally or compositionally from the development data, it could affect the type of validation, consequently affecting performance [35]. This underscores the importance of transparent validation methods to ensure consistent predictive accuracy across datasets. Additionally, while the ANN was found to slightly overestimate the risk of 30-day readmission in patients undergoing primary TKA, the SRC underestimated readmission risk. In the current context, the slight overestimation of risk may be preferable in a clinical decision model compared with an underestimation of risk, since episodes of readmission following surgery have been shown to negatively affect patient satisfaction and functional recovery [6, 7], as well as overall healthcare expenditure [4, 5]. Furthermore, the ANN model was developed using only the variables included in the SRC to ensure a consistent comparison between the two predictive models. However, the variable selection for the SRC may have been influenced by the logistic regression-based approach used, potentially overlooking other important predictors that may have been identified by an ML-based approach. Therefore, the predictive performance of the ANN model developed in this study could be significantly improved if the predictors were optimally selected for ML-based modeling using an established technique of recursive feature elimination [20, 43]. Additional studies are warranted to further optimize the ANN model to assist decision-making in clinical settings.

We found that the body mass index (BMI) was the most important determinant of readmission following surgery. A BMI greater than 34 kg/m^2^ was associated with an increase in the risk of readmission after primary TKA. Previous studies have reported similar findings, with higher BMI associated with an increase of risk of readmission after primary TKA [44, 45]. In an analysis conducted using the ACS NSQIP database, George et al. found that BMI demonstrated a U-shaped relationship with 30-day readmission, with the lowest readmission rates occurring at a BMI closer to 30 kg/m^2^. [45]. The findings of our study are similar in this regard. However, when BMI was analyzed as a categorical variable, the study found that only morbid obesity (BMI > 40 kg/m^2^) was associated with a statistically significant risk of being readmitted after surgery. Several studies analyzing BMI as a categorical variable previously have reported similar findings [44, 46]. The consideration of BMI as a categorical variable, therefore, may not offer as valuable an insight into readmission risk as the inclusion of BMI as a continuous variable. The findings of the current study may assist in informing an optimal BMI target for preoperative optimization in patients undergoing total knee arthroplasty. Our study also found advanced age (> 69 years) to be an important predictor of 30-day readmission. Older age has been shown to be associated with an increased risk of readmission in previous analyses [46, 47]. While age is a nonmodifiable risk factor, our findings further underscore the importance of increased postoperative monitoring in older patients to mitigate the occurrence of unplanned readmission. The current study also found the male sex to be a strong predictor of readmission after TKA, in accordance with the existing literature. Men have been shown to be at a higher risk of readmission following TKA in several previous studies [46, 48–50]. Although the exact cause of this is still unclear, it has been hypothesized to be owing to relatively lower estrogen levels present in men, thereby precluding them from the protective effect of estrogen on bone health and joint recovery [49]. Alternately, men may be at an increased risk of readmission due to their higher risk of developing serious postoperative complications such as surgical site infection or cardiac arrest [48]. Nevertheless, the identification of predictors strongly associated with readmission may assist during preoperative counseling of at-risk patients and shape appropriate postoperative monitoring strategies in these patient subgroups.

The results of our study must be interpreted in the context of their limitations. The ACS-NSQIP only records readmission up to 30 days following surgery, which may not adequately represent all episodes of readmission associated with surgery. Recent evidence suggests that 90-day outcomes may be more appropriate measures of surgical quality [51, 52]. Efforts should be made to include 90-day outcomes in national databases such as the ACS-NSQIP to better inform quality improvement interventions. Additionally, the observed results may be influenced by potential differences between the models being compared (Type 2a versus Type 3). While beyond the scope of this study, future research should consider external validation of both models in other independent datasets to improve comparison consistency. Furthermore, the size of the validation cohort in the current study was limited by the number of manual entries of patient data into the SRC, as the ACS-NSQIP does not allow automation of its surgical risk calculator. Future emphasis on technology sharing and integration may allow better evaluation of existing risk prediction tools, potentially improving patient outcomes after surgery. Finally, to ensure consistent comparison, the ANN model in this study was developed using only the variables included in the SRC. However, other factors that could influence readmission risks—such as social, economic, environmental variables, and preoperative lab investigations—were not considered in the current study and merit further investigation.

## Conclusions

This study demonstrates the superior predictive performance of machine learning algorithms, such as the ANN, compared with the conventional ACS-NSQIP surgical risk calculator when developed using the same set of predictor variables. Although the predictive performance of the ANN developed in this study may be subsequently improved by including other important input parameters, the ANN model identified BMI greater than 34 and age over 69 years as the most important predictors of readmission following primary TKA. The findings of our study may facilitate the development of novel machine learning-based clinical decision support tools, potentially improving patient-specific preoperative counseling and postoperative monitoring practices in at-risk patients.

## Data Availability

The datasets generated and/or analyzed during the current study are not publicly available due to the ACS-NSQIP data sharing agreement, but are available for download directly from the ACS-NSQIP for participating institutions.

## Abbreviations

TKA: Total knee arthroplasty
ACS-NSQIP: American College of Surgeons National Surgical Quality Improvement Program
SRC: Surgical risk calculator
ML: Machine learning
CPT: Current procedural terminology
ANN: Artificial neural network
BMI: Body mass index
ASA: American Society of Anesthesiologists
